# Promising Outcomes of Modified ALPPS for Staged Hepatectomy in Cholangiocarcinoma

**DOI:** 10.3390/cancers15235613

**Published:** 2023-11-28

**Authors:** Arianeb Mehrabi, Mohammad Golriz, Ali Ramouz, Elias Khajeh, Ahmed Hammad, Thilo Hackert, Beat Müller-Stich, Oliver Strobel, Sadeq Ali-Hasan-Al-Saegh, Omid Ghamarnejad, Mohammed Al-Saeedi, Christoph Springfeld, Christian Rupp, Philipp Mayer, Markus Mieth, Benjamin Goeppert, Katrin Hoffmann, Markus W. Büchler

**Affiliations:** 1 Department of General, Visceral and Transplantation Surgery, University of Heidelberg, 69120 Heidelberg, Germany; 2 Liver Cancer Center Heidelberg (LCCH), Heidelberg University Hospital, 69120 Heidelberg, Germany; 3 Department of Medical Oncology, National Center for Tumor Diseases, Heidelberg University Hospital, 69120 Heidelberg, Germany; 4 Department of Internal Medicine, Heidelberg University Hospital, 69120 Heidelberg, Germany; 5 Department of Interventional Radiology, Heidelberg University Hospital, 69120 Heidelberg, Germany; 6 Institute of Pathology, Heidelberg University Hospital, 69120 Heidelberg, Germany

**Keywords:** cholangiocarcinoma, future liver remnant volume, ALPPS, posthepatectomy liver failure

## Abstract

**Simple Summary:**

The aim of the current study was to evaluate the outcomes of associating liver partition and portal vein ligation (ALPPS) in patients with cholangiocarcinoma and the effect of technique modification in this regard. In this observational study, modified ALPPS reduced the morbidity and mortality rates in patients with cholangiocarcinoma, particularly with perihilar cholangiocarcinoma. Compared with the literature, a minimized stage one procedure contributes to diminished pre-stage two risk score, and it improves the posthepatectomy outcomes. In patients with cholangiocarcinoma, the modified ALPPS, by using a minimized stage one procedure, should be considered to be a safe and beneficial curative procedure to enhance patients’ survival.

**Abstract:**

Associating liver partition and portal vein ligation for staged hepatectomy (ALPPS) is a two-stage procedure that can potentially cure patients with large cholangiocarcinoma. The current study evaluates the impact of modifications on the outcomes of ALPPS in patients with cholangiocarcinoma. In this single-center study, a series of 30 consecutive patients with cholangiocarcinoma (22 extrahepatic and 8 intrahepatic) who underwent ALPPS between 2011 and 2021 was evaluated. The ALPPS procedure in our center was modified in 2016 by minimizing the first stage of the surgical procedure through biliary externalization after the first stage, antibiotic administration during the interstage phase, and performing biliary reconstructions during the second stage. The rate of postoperative major morbidity and 90-day mortality, as well as the one- and three-year disease-free and overall survival rates were calculated and compared between patients operated before and after 2016. The ALPPS risk score before the second stage of the procedure was lower in patients who were operated on after 2016 (before 2016: median 6.4; after 2016: median 4.4; *p* = 0.010). Major morbidity decreased from 42.9% before 2016 to 31.3% after 2016, and the 90-day mortality rate decreased from 35.7% before 2016 to 12.5% after 2016. The three-year survival rate increased from 40.8% before 2016 to 73.4% after 2016. Our modified ALPPS procedure improved perioperative and postoperative outcomes in patients with extrahepatic and intrahepatic cholangiocarcinoma. Minimizing the first step of the ALPPS procedure was key to these improvements.

## 1. Introduction

Cholangiocarcinomas are the second most common primary hepatic malignancy, accounting for around 10% of the hepatobiliary tumors [1,2]. Over the past two decades, the incidence of cholangiocarcinoma has increased worldwide. Overall, cholangiocarcinoma has a very poor prognosis with a five-year survival rate of 5–10%. Surgery can increase the five-year survival rate to 25–30%, but 60–90% of patients are considered unresectable, because their disease is too extensive or their functional future remnant liver volume (RLV) is insufficient [3,4,5,6,7].

Recent developments in surgical techniques and perioperative patient management such as portal vein embolization (PVE), two-stage hepatectomy, and associating liver partition and portal vein ligation for staged hepatectomy (ALPPS) have addressed the problems of insufficient functional RLV volume and poor postoperative outcomes [5,8]. Of these methods, ALPPS is considered to be a modern and complex therapeutic approach for the treatment of malignant liver tumors [9], which gives the best results in terms of the interstage dropout rate, future RLV hypertrophy, and resection rate [6,10,11,12]. Although cholangiocarcinoma is not considered anymore to be an uncommon indication for ALPPS [13], the superiority of ALPPS has not been conclusively determined in patients with cholangiocarcinoma [12,14,15,16,17]. A recent multicenter study showed that surgical procedure refinements in ALPPS improved the survival of patients with intrahepatic cholangiocarcinoma (IHCC) [18]. However, the morbidity and mortality rates in these patients remain high following ALPPS, leading some surgeons to believe that ALPPS should be contraindicated in patients with cholangiocarcinoma, particularly perihilar cholangiocarcinoma (PHCC). Although IHCC and PHCC are two different entities regarding management and prognosis, PHCC and IHCC with biliary compression could be approximated due to aggressiveness and clinical image.

We have been treating cholangiocarcinoma patients who were considered unresectable because of insufficient RLV with the ALPPS procedure since 2011. Considering the current doubt regarding the ALPPS in the treatment of patients with cholangiocarcinoma, in the present study, we report our encouraging single-center experience with ALPPS in these patients. Additionally, in 2016, we minimized the first step of the ALPPS procedure in an attempt to improve the postoperative outcomes. We believe that this modification can also reduce the individual risk of patients before the second stage of ALPPS, according to the ALPPS risk score [19]. The current study focuses on the modification and management approach, which aimed to increase the resectability and reduce the morbidity in patients with unresectable cholangiocarcinoma. Thus, the outcomes of the patients before and after modification of the procedure and management will be also compared.

## 2. Materials and Methods

### 2.1. Study Population

Seventy ALPPS procedures were performed in our center between October 2011 and June 2021. Thirty of these were performed on patients with cholangiocarcinoma. Peri- and postoperative data were collected from our prospectively maintained database. Therapeutic decisions were made by an interdisciplinary institutional liver board that included surgeons, hepatologists, oncologists, radiooncologists, and pathologists. All patients underwent computed tomography (CT) or magnetic resonance imaging to assess the resectability of the tumors and the functional RLV. Patients without lymph nodes or distant metastases, who could not receive a complete tumor resection due to tumor burden, were classified as primary unresectable [20]. Accordingly, patients with potentially an RLV < 30% were assessed for the ALPPS procedure [20,21,22,23], while preoperative portal vein embolization was carried out in none of the patients. The timing of second stage was decided with due attention to the adequacy of liver hypertrophy via CT volumetry. In an interval of approximately seven days after the first stage, all patients underwent computed tomography (CT) and subsequent liver volumetry to prove the sufficiency of RLV (≥30% of the primary liver volume [23,24]). The second stage was planned immediately for patients with adequate liver hypertrophy, whereas patients with insufficient RLV underwent second CT-volumetry at the end of second week after the first stage.

The study protocol was approved by the independent ethics committee of the University of Heidelberg (approval number: S-754/2018), and it is also registered in the ISRCTN registry with the registration number ISRCTN10972467. This study is compliant with the STROCSS criteria (strengthening the reporting of cohort studies in surgery), and the respected checklist has been provided in the Supplementary Material [25]. 

### 2.2. Change Management and ALPPS Modification

In 2016, we started using the ALPPS risk score to preoperatively assess patients for the ALPPS procedure [19,26]. In our center, patients with tumor-related bile duct obstruction and severe hyperbilirubinemia (serum bilirubin > 15 mg/dL) are not primarily eligible for surgical procedure and are thus candidates to undergo preoperative biliary drainage to resolve the bile stasis and decrease the serum levels of bilirubin. In these patients, endoscopic retrograde cholangiopancreatography (ERCP) or stent placement was carried out to decrease the serum bilirubin level. Percutaneous transhepatic bile duct drainage (PTBD) was indicated in patients after failure of the ERCP attempt.

In the ALPPS procedure, an intraoperative ultrasound examination was carried out after complete mobilization of the liver to exclude undetected metastases. Afterward, the hepatic hilum was dissected, and the right portal vein was prepared and divided by a stapler device. Subsequently, the right hepatic artery and the right bile duct were exposed and marked with a vessel loop. The right hepatic vein was also dissected and marked with a vessel loop. Afterward, a hanging maneuver was performed and anatomical parenchymal dissection (partial or complete based on the surgeons’ discretion) was performed by LigaSure, accordingly. We also modified our standard ALPPS procedure by minimizing the first stage of the surgical procedure [19,27]. The specific modifications during the first stage involved delaying biliary reconstruction and the externalization of the biliary flow. Despite patients with serum bilirubin >15 mg/dL, other patients with jaundice, biliary congestion, and hyperbilirubinemia, who had a serum bilirubin level of <15 mg/dL, were eligible for ALPPS without preoperative biliary drainage. However, to decompress the proximal bile duct and reduce the risk of cholangitis in the interstage phase, the reconstruction of the biliary tract is postponed to the second stage and the externalization of the biliary flow is utilized. To externalize the biliary flow, a pediatric feeding tube was inserted into the proximal bile duct. During the interphase stage, antibiotics were administered in all the patients. Biliary reconstructions were performed during the second stage.

### 2.3. Patient Data Collection

*Preoperative evaluations:* Preoperative clinical data, including demographic data, laboratory test results, cholangiocarcinoma type (IHCC and PHCC), and the ALPPS risk score were recorded before the ALPPS procedure.

*Stage 1 intraoperative evaluations:* Intraoperative data were collected during the first stage of the ALPPS procedure. This included the type of liver resection according to the Brisbane 2000 nomenclature [28], operation time, blood loss, and whether a packed red blood cell (RBC) transfusion was performed.

*Interstage evaluations:* Data were also collected before the second stage of the ALPPS procedure. These included results from CT imaging and liver volumetry. The RLV was measured using 3D volumetric software AquariusNET 4.4 (TeraRecon, Inc., Foster City, CA, USA) after uploading the DICOM files of postoperative CT images (portal phases) to the local server. The ALPPS risk score was calculated for the second stage of the procedure.

*Stage 2 intraoperative evaluations:* Intraoperative data were collected during the second stage of ALPPS procedure, including operation time, blood loss, and whether a packed RBC transfusion was performed.

*Postoperative evaluations and follow-up:* The tumor TNM staging and grading, as well as the resection margin (R0/1), were reported. Postoperative morbidities were classified as grade I to V based on the Clavien–Dindo classification [29]. Major morbidity was defined as grade III or IV. Posthepatectomy liver failure (PHLF) was diagnosed and reported according to the International Study Group of Liver Surgery (ISGLS) [30]. PHLF was graded as A, B, or C. Postoperative mortality was defined as all-cause death occurring within the first 90 days after surgery. Overall survival (OS) was defined as the time from the operation to the date of death or last follow-up. Recurrence-free survival (RFS) was defined as the time to recurrence or death event after surgery. OS and RFS were monitored for three years.

### 2.4. Statistical Analysis

Statistical analysis was performed using IBM SPSS Statistics for Windows, version 27.0 (IBM Corp. Released 2013. Armonk, NY, USA). Categorical data were presented as frequencies and proportions, and continuous data were presented with the median and range except for liver volume calculations, which were provided as mean ± standard deviation. Categorical data were compared using Chi-square test of association or Fisher’s exact test. A Mann–Whitney test was conducted to determine if there was a shift of the distribution of pre- and postoperative continuous data between patients with different outcomes. The Kaplan–Meier method was used to estimate survival curves, and the log-rank test was used to compare the survival outcomes between patients having undergone ALPPS before and after 2016. A two-sided *p* value less than 0.05 was considered significant in all analyses.

## 3. Results

### 3.1. Preoperative Data

The median age of patients was 67 years (range: 39–81) and 24 patients (80%) were male. PHCC was the most common indication for ALPPS (22 patients [73.3%]) and the median CA 19-9 tumor marker value was 73.7 U/mL. The median interval between the diagnosis of the disease and the first stage of ALPPS was 24 days (range: 6–223). The median preoperative serum bilirubin level was 1.7 mg/dL the day before operation. The median preoperative internal normalized ratio (INR) was 1.00 and the median preoperative serum albumin was 40 g/L. Before the first stage, the mean preoperative RLV was 301.6 ± 74.8 cm^3^ (21.6 ± 5.7% of the whole liver) and the median ALPPS risk score was 3.5. The ALPPS risk score before the first stage did not change significantly after the surgical modifications were introduced (before 2016: 2 (2–5); after 2016: 2 (2–5); *p* = 0.552). The preoperative data are presented in Table 1.

### 3.2. Stage 1 Intraoperative Data

All patients underwent right hepatectomy; 21 patients received an extended hepatectomy and 9 patients underwent hemihepatectomy. Right hepatectomy was indicated in two patients with type IIIB Klatskin tumors due to the infiltration of the right hepatic artery. The median blood loss during the first stage of the procedure was 600 mL. Two patients (6.7%) needed an RBC transfusion during the first stage of the procedure. The median surgical time was 266 min (range: 127–570 min) (Table 2). After 2016, we did not perform hepaticojejunostomy during the first stage in patients with hyperbilirubinemia (serum bilirubin < 15 mg/dL) and received no preoperative interventions. These patients received a temporary external drainage into the bile duct that remained in place until the second stage of the operation. This significantly reduced the operating time of the first stage (from a median of 337 min before 2016 to 240 min after 2016; *p* = 0.01). No intraoperative complications were reported during the first stage of the procedure.

### 3.3. Interstage Data

The median interval between the two stages was 8 (range: 6–26 days) days. The mean RLV after liver hypertrophy before the second stage was 625.1 ± 111.6 cm^3^ (46.5 ± 0.07% of the whole liver), and the mean kinetic growth rate was 15% per day. In patients who underwent the ALPPS procedure before 2016, the median serum bilirubin levels were 1.7 mg/dL and 1.4 mg/dL before stages I and II, respectively (*p* = 0.48). Following modifications to the ALPPS procedure after 2016, there was a significant decrease in the median serum bilirubin levels from 2.6 mg/dL before the first stage to 1.0 mg/dL before the second stage of ALPPS (*p* = 0.03). All patients underwent the second stage of the ALPPS procedure. Postoperative major complications occurred in 6.7% of patients (*n* = 2) after the first stage (Table 3). 

These complications included hemorrhage (venous bleeding from the resection plate) and dislocation of the temporary biliary drain, which were managed with surgical revision. Major morbidity after the first stage was seen in one patient (1/14; 7.1%) before 2016 and in one patient after 2016 (1/16; 6.3%) (*p* = 0.956). No mortality was reported during the interval between the two stages. The ALPPS risk score before stage 2 was significantly lower between patients who were operated after 2016 compared to that of before 2016 (4.4 vs. 6.4; *p* = 0.010) (Table 4).

### 3.4. Stage 2 Intraoperative Data

During the second stage, the median blood loss was 600 mL. An RBC transfusion was needed in four patients (13.3%) and the median operating time was 155 min (range: 60–339 min). After 2016, the hepaticojejunostomy was performed during the second stage of the ALPPS procedure in all patients. The median operating time increased significantly from 97 min before 2016 to 207 min after 2016 (*p* = 0.002). No intraoperative complications were reported during the second stage (Table 3).

### 3.5. Postoperative Outcomes

Major complications occurred in 11 patients (36.7%) after the second stage of the ALPPS procedure. These complications included bile leakage (*n* = 5), burst abdomen (*n* = 3), and colon ischemia with perforation (*n* = 3). Of the five patients with biliary leakage, three had leakage from the anastomosis and two from the resection plane. Of these, one patient received CT drainage, and surgical revision was needed in four cases. One patient received percutaneous transhepatic bile duct drainage (PTBD) after surgical revision because of constant leakage. The median hospital stay was 43.5 days (range: 16–101 days), and the 30-day postoperative mortality was 6.7% (*n* = 2)—one patient died of necrotizing pancreatitis and the other patient died of multiple organ failure (MOF) following grade C PHLF. The 90-day postoperative mortality was 23.3% (*n* = 7)—four patients (13.3%) died of MOF following grade C PHLF, two (6.7%) died of sepsis, and one (3.3%) died of necrotizing pancreatitis (Table 3).

The ALPPS modifications were introduced in 2016 after change management in our center, which reduced the pre-stage 2 ALPPS risk score. The major postoperative morbidity reduced from 42.9% before 2016 to 31.3% after 2016, but this difference was not statistically significant (*p* = 0.390). The ALPPS risk score before stage 2 was significantly lower in patients who did not develop severe postoperative morbidity (*p* = 0.031). In addition, 90-day mortality decreased remarkably from 35.7% before 2016 to 12.5% after 2016, although this difference was not statistically significant (*p* = 0.197) (Table 4). ALPPS risk scores before stage 2 were significantly lower in patients who did not die during the first 90 postoperative days (*p* = 0.025).

### 3.6. Histopathological Results

The final histopathological results showed that 23 patients (76.7%) had T2 tumors. T4 and T3 tumors were detected in four (13.3%) and three (10%) patients, respectively. In 63.3% of the patients, no lymphatic metastasis was reported. Regional lymph node infiltration (N1) was reported in 33.3% of the patients. N2 was detected in only one patient (3.4%). None of the patients suffered from distant metastasis. Twenty-five patients (83.3%) had moderately differentiated tumors (G2). Low and highly differentiated tumors were reported in two (6.7%) patients and one (3.3%) patient, respectively, and two patients (6.7%) had unknown grading status. Twenty-six patients (86.7%) underwent an R0 resection, which showed no significant difference before and after 2016 (*p* = 0.552).

### 3.7. Oncological Long-Term Outcomes

**Figure 1:**
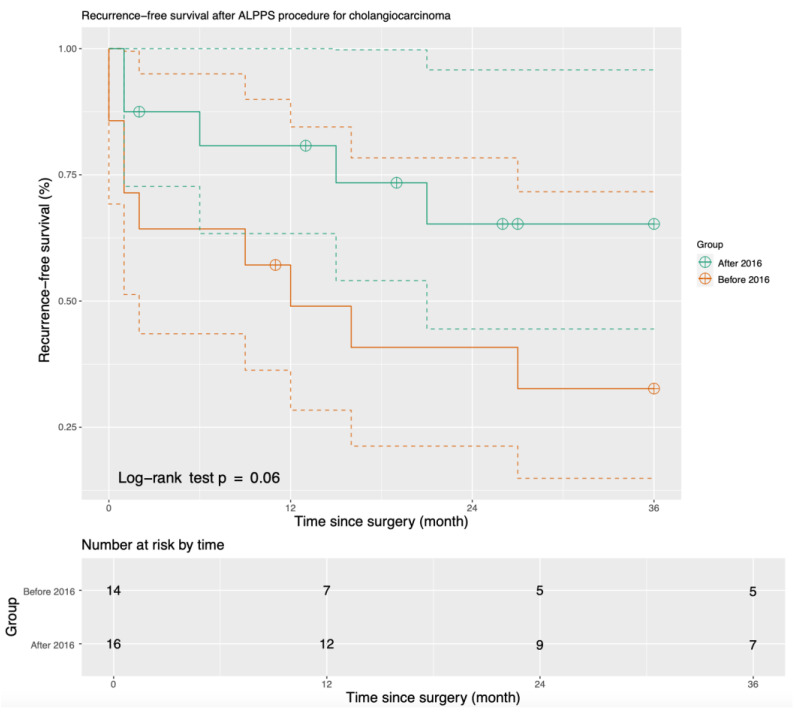
Three-year recurrence-free survival of patients who underwent ALPPS (Solid lines present the actual survival curve, and dash lines define the confidence intervals).

**Figure 2:**
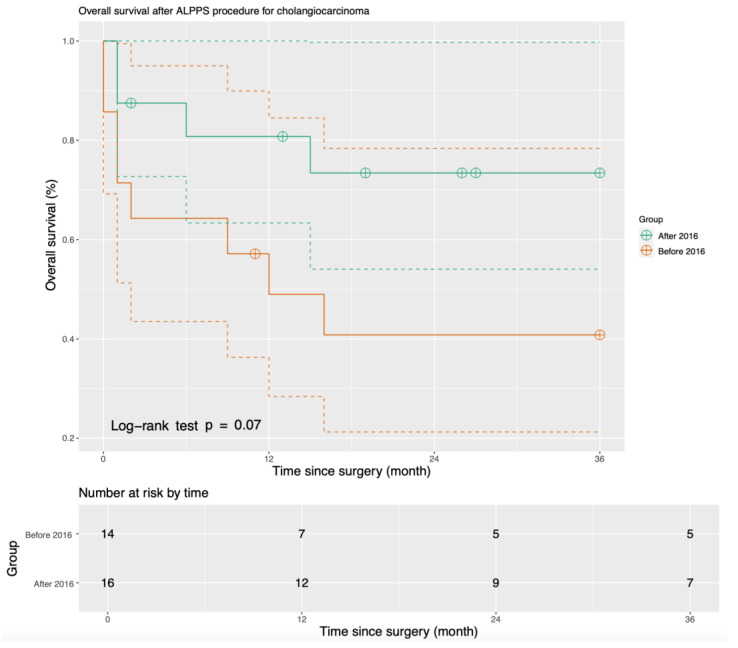
Three-year overall survival of patients who underwent ALPPS (Solid lines present the actual survival curve, and dash lines define the confidence intervals).

The median duration of follow-up after the ALPPS procedure was 31 months (range 0–127 months). RFS and OS both increased after we modified our ALPPS procedure in 2016. The one-year OS increased from 40.9% before 2016 to 80.8% after 2016. The three-year RFS increased from 30.7% before 2016 to 65.3% after 2016 (*p* = 0.06) (Figure 1), and the three-year OS increased from 40.8% before 2016 to 73.4% after 2016 (*p* = 0.07) (Figure 2).

To highlight the oncological distinctions between these two conditions, subgroup survival analyses were conducted for patients with PHCC. Similar to the whole cohort of the study, RFS and OS both increased in patients with PHCC after the modification of ALPPS. Albeit not significant, the three-year RFS increased from 33.3% before 2016 to 63.6% after 2016 (*p* = 0.11) (Supplementary Figure S1). The three-year OS was 33.3% before 2016, which increased to 72.7% after 2016 (*p* = 0.16) (Supplementary Figure S2).

## 4. Discussion

Cholangiocarcinomas have a very poor prognosis [31]. Curative surgery prolongs survival, but many patients with cholangiocarcinoma are not eligible for surgical resection because they have insufficient RLV [32,33]. The ALPPS procedure has been shown to overcome the problem of resection in patients with insufficient RLV with a high successful resection rate [8,34,35] and has improved long-term oncologic results in patients with colorectal liver carcinoma [12,36]. Currently, ALPPS is not considered to be an uncommon procedure for patients with cholangiocarcinoma [13]. However, whether ALPPS can improve survival in cholangiocarcinoma patients is not well defined [34,37,38], and many surgeons are unwilling to consider ALPPS as a surgical treatment option for cholangiocarcinoma because of the associated high perioperative morbidity and mortality. Considering other techniques, a recent study by Falken et al. suggested that using portal vein embolization might remarkably decrease the liver failure and mortality rates [39]. Although they reported a PHLF rate of 4% followed by a mortality rate of 2%, it must be mentioned that in this study, the authors have proposed a four-fold increase for the cut-off value of future remnant liver function prior to liver resection. However, many patients with advanced cholangiocarcinoma, who suffer from severe liver damages, might not fulfill the defined criteria. In these cases, ALPPS can still be taken into account for curative tumor resection and for preventing PHLF [18,40].

Many attempts have been made to modify the ALPPS procedure to make it more suitable for treating patients with cholangiocarcinoma, including technical modifications and better patient selection [12,41,42,43,44,45,46,47]. These modifications all aimed to minimize the first stage of the surgical procedure as much as possible. Particularly among patients with PHCC, hyperbilirubinemia due to bile duct obstruction is associated with infections, PHLF, intraoperative blood loss, and renal insufficiency [48,49]. Therefore, preoperative biliary drainage via ERCP and PTBD is considered to be a bridging treatment to prevent adverse postoperative outcomes. However, it has been shown that ERCP and PTBD are not entirely harmless procedures, considering the increased risk of tumor seeding, prolonged hospital stays, morbidity, and infections among patients [50,51,52]. Therefore, we limited preoperative biliary drainage to patients with a serum bilirubin level exceeding 15 mg/dL, considering the higher risk of liver failure after ALPPS in these patients. Intraoperative decompression of the biliary tract and externalization of biliary outflow were carried out during the first stage of ALPPS for patients with a bilirubin level below 15 mg/dL. However, externalization of biliary flow facilitates the remission of liver function and avoids the endotoxemia [53]. Recent studies have demonstrated that functional and volumetric hypertrophy were similar after the first stage of both conventional and modified ALPPS; therefore, modified ALPPS is preferred because it lowers morbidity and mortality without compromising other treatment outcomes [10,54]. Although a direct comparison between different modifications of ALPPS, such as partial ALPPS, radiofrequency-assisted ALPPS, and associating liver tourniquet and portal vein ligation for staged hepatectomy (ALTPS) was not possible due to not enough available data, a recent meta-analysis revealed similar outcomes from different techniques, using a single-arm meta-analysis [54]. Furthermore, considering the complexity of the ALPPS procedure and the importance of the precise patients’ selection, the ALPPS risk score was developed to identify patients at risk of early ALPPS-related morbidity and mortality [19,26,55]. In line with these findings, we performed a change management and introduced modifications to our surgical ALPPS procedure to improve the postoperative outcomes. These modifications led to reduced operation time in the first stage of ALPPS, as well as increased operation time in the second stage. By minimizing the surgical intervention in the first stage of the ALPPS procedure, we could reduce the risk to the patients as measured by the ALPPS risk score before the second stage of the procedure. 

In a recent study, Li et al. [18] reported that ALPPS resulted in better oncological outcomes by providing a more efficient free-margin resection in patients with locally advanced IHCC. However, the efficacy of the ALPPS procedure in treating PHCC remains unclear, and PHCC is still considered a contraindication for ALPPS because of its associated high mortality [39]. For example, Olthof et al. [14] reported a 90-day mortality rate of 48% following ALPPS in 29 PHCC patients compared with a 90-day mortality rate of 13% following conventional methods of major liver resection in 29 matched patients. However, in this study, the ALPPS patients were selected from 37 ALPPS procedures performed over only 4 years while the matched group were selected from 257 conventional procedures performed over 16 years. Therefore, the higher surgical experience regarding the extended liver resections might be considered to be a factor leading to better outcomes in patients undergoing extended hepatectomy in their study. In our present study, 73.3% of included patients had PHCC, and we showed promising outcomes with a 90-day mortality rate of 12.5% after modification of the technique and the management approach, as well as improvements in the short- and long-term oncological outcomes. The 90-day mortality rate was 12.5% after 2016, which was in the range of mortality rates after extended resection for PHCC at western centers (6.5–33.3%) and which was even lower compared to the rates reported for patients in these series with RLV less than 30% (>26.5%) [56,57]. Nonetheless, patients undergoing ALPPS in the current survey were suffering from primarily unresectable tumors. 

In addition, our outcomes were improved even further after we minimized the first stage of the procedure. After 2016, the ALPPS risk score reduced significantly in our patients before stage 2, which in turn resulted in better postoperative short- and long-term outcomes. These findings suggest that PHCC should not be considered as a contraindication for ALPPS anymore, and that a modified ALPPS procedure might be able to improve the surgical outcomes in patients with PHCC. The 90-day mortality rates we observed following the use of ALPPS for cholangiocarcinoma were better than those reported in the literature [14], particularly in PHCC patients. A postoperative major complication rate of 31% and a 90-day mortality rate of 12.5% were observed after modifying our ALPPS procedure in 2016. Truant et al. [20] reported a major complication rate of 40.3% and a mortality rate of 12.9% in a large multicenter study of nine high-volume hepatobiliary centers with 62 patients who underwent ALPPS. Of note, 80.6% of patients in the Truant et al. study had colorectal liver metastasis and only 42.4% underwent extended hemihepatectomy via ALPPS [20]. Two other studies have reported ALPPS outcomes from the ALPPS registry: Schadde et al. and Schnitzbauer et al. [34,58]. In the study of Schadde et al., a major complication rate of 40.0% and a mortality rate of 9.4% were reported in 202 patients who underwent ALPPS [34]. In their study, 70.0% of patients had colorectal liver metastasis and only 42.6% underwent extended hemihepatectomy. They reported a 90-day mortality rate of 27% in 11 patients with PHCC. Schnitzbauer et al. reported a major complication rate of 11.9% and a mortality rate of 7.2% in 403 patients from the ALPPS registry [58]. All these patients had colorectal liver metastasis and 54.6% of them underwent extended hemihepatectomy. In contrast to these published studies, 73% of our patients had PHCC and 70% underwent an extended liver resection (resection of ≥five liver segments). Considering this higher proportion of extended resections, a higher complication rate would be expected; however, we observed promising results. Li et al. [18] were the first to show that ALPPS can produce acceptable outcomes in patients with IHCC. In the present study, we support the idea of pushing the limits of ALPPS even further by showing that ALPPS reaches promising results in patients with PHCC [59]. However, it should be taken into consideration that major and aggressive procedures should be carried out in a high-volume hepatobiliary center, while standardized perioperative care protocols as well as a center’s experience are the main factors affecting the postoperative outcomes, in addition to surgical skill [60,61].

Patients with cholangiocarcinoma who underwent conventional liver resection were reported to have the median RFS and OS of 24.4 and 51.1 months, respectively [62]. The median OS in our cohort was 31 months. Due to low future RLV and tumor burden, these patients were considered to be unresectable and conventional liver resection was not affordable in these patients. Thus, excluding the ALPPS procedure, no curative treatment was achievable and palliative treatment would be the only therapeutic option. In these settings, we believe the median OS of 31 months after ALPPS should be compared to that of 11.7 months (range 6.9–12.3 months) in patients undergoing palliative treatment, which reveals the superiority of survival after ALPPS [62,63,64,65]. Among patients with inoperable CC undergoing systemic therapies, the one-year and three-year OS rates were 18% and 3%, respectively [66], which were considerably lower compared to 73% of three-year OS after using the modified ALPPS in the current study. However, the oncological outcomes should be interpreted in the light of the fact that the ALPPS have impacted the patients’ survival, since patients died due to PHLF or other major complications, whereas they could be alive at 90 days without surgery. On the other hand, it should be taken into account that a successful ALPPS procedure in patients with unresectable tumors is capable of improving the patients’ long-term oncological outcomes as well as survival. There is little evidence available in the literature concerning the application of adjuvant chemotherapy after liver resection for cholangiocarcinoma and the respected outcomes. The American Society of Clinical Oncology (ASCO) guidelines suggest adjuvant capecitabine chemotherapy for a duration of six months in patients who have undergone liver resection for cholangiocarcinoma [67]. Furthermore, the guidelines suggest a combined chemoradiotherapy in patients with extrahepatic cholangiocarcinoma with a positive surgical resection margin [67]. Despite recommendation of the ASCO guidelines based on the BILCAP trial, the role of adjuvant chemotherapy after liver resection in cholangiocarcinoma remains controversial and under debate [68,69]. Therefore, the decision regarding the postoperative management of patients should be made in multidisciplinary sessions for a comprehensive assessment of advantages and disadvantages. 

With due attention to the latest developments of the ALPPS in the recent years, there is an urgent need for defining the reference values for relevant outcome parameters of this procedure for different indications [70,71,72]. Recently, a study published by Raptis et al. defined the benchmark ALPPS outcomes in patients with colorectal liver metastases [72]. Some of these defined benchmarks were a stage two completion rate of ≥96%, PHLF after stage 2 ≤ 5%, combined two-stage major morbidity and 90-day mortality rates of ≤38% and ≤5%, respectively, and one-year DFS of ≥50%. Although in our study, PHCC was the most common indication (73%), the surgical technique’s success rate was 100% with a median interstage interval of 7 days. Major complications and 90-day mortality rates in our study were 36% and 23%, respectively. Despite the fact that our study was carried out among patients with cholangiocarcinoma, except for the mortality rate and PHLF, our results were in the ranges of the proposed benchmarks. 

Our study has some limitations. The main limitations of the present study are the number of patients and the non-randomized study design. A large multicenter randomized-controlled trial is needed to confirm our findings. Given the small sample size, no multivariate analysis was carried out in order to prevent presentation of unreliable outcomes. However, some differences in the distribution of potential prognostic factors between two groups (before 2016 and after 2016), such as preoperative biliary drainage, could have affected the results as cofounding bias. Furthermore, the experience of the interdisciplinary team in the selection of patients and in carrying out the ALPPS procedure could be considered to be another cofounding bias. Although randomized controlled trials are needed to evaluate the efficacy of ALPPS in the treatment of patients with cholangiocarcinoma, it might not be realistic when considering the very limited pool of patients. Therefore, future studies should focus on prioritizing a trial of ALPPS versus PVE or single stage resection in patients with PHCC.

## 5. Conclusions

The ALPPS procedure was modified by externalizing the biliary flow, delaying biliary reconstruction to the second stage, and employing interstage antibiotic therapy. Our experience of extended liver resection strongly suggests that using the modified ALPPS procedure in patients with cholangiocarcinoma with insufficient RLV produces better outcomes, particularly in patients with PHCC. The key to this improvement was minimizing the first stage of the operation to reduce patient risk during the second stage. However, future studies are needed to define benchmarks for the ALPPS procedure among cholangiocarcinoma patients. 

## Figures and Tables

**Table 1:** Characteristics of patients who underwent ALPPS.

Variable of Interest	All Patients (*n* = 30)
Age (range)	67 (39–81)
Gender	
Female/male	6/24
BMI (kg/m^2^) (range)	24.9 (19.04–29.70)
CA 19-9 (U/mL) (range)	73.7 (8.8–4044.0)
Tumor type	
PHCC, *n* (%)	22 (73.3%)
Bismuth Corlette type II	1 (3.3%)
Bismuth Corlette type IIIA	4 (13.3%)
Bismuth Corlette type IIIB	2 (6.7%)
Bismuth Corlette type IV	15 (50%)
IHCC, *n* (%)	8 (26.7%)
Median INR before stage 1 (range)	1.0 (0.71–4.58)
Median serum albumin before stage 1 (g/L) (range)	40 (24.2–47.4)
Preoperative biliary stenting, *n* (%)	5 (18.5%)
Median serum total bilirubin before stage 1 (mg/dL) (range)	1.7 (0.3–12.1)
Median serum total bilirubin before stage 2 (mg/dL) (range)	1.2 (0.2–11.0)
Median serum creatinine before stage 2 (g/L) (range)	0.69 (0.5–2.1)
ALPPS risk score before stage 1, median (range)	3.5 (2–5)
ALPPS risk score before stage 2, median (range)	5.5 (3.1–9.6)
Median RLV/whole liver before stage 1 (%) (range)	22 (15–32)
Median RLV/whole liver before stage 2 (%) (range)	47 (29.1–58)

ALPPS: associating liver partition and portal vein ligation for staged hepatectomy; BMI: body mass index; CA 19-9: cancer antigen 19-9; INR: international normalized ratio; IHCC: intrahepatic cholangiocarcinoma; PHCC: perihilar cholangiocarcinoma; RLV: remnant liver volume; SD: standard deviation.

**Table 2:** Intraoperative data of patients who underwent ALPPS.

Variable of Interest	All Patients (*n* = 30)
Type of resection *n* (%)	
Right hepatectomy	9 (30.0%)
Right trisectionectomy	21 (70.0%)
ALPPS stage 1	
Median operation time (minutes) (range)	266 (127–570)
Blood loss ALPPS stage 1 (mL) (range)	600 (200–4000)
Patients transfused with RBC, *n* (%)	2 (6.7%)
ALPPS stage 2	
Median operation time (minutes) (range)	155 (60–339)
Blood loss (mL) (range)	600 (100–3500)
Patients transfused with RBC, *n* (%)	4 (13.3%)

ALPPS: associating liver partition and portal vein ligation for staged hepatectomy; RBC: red blood cell.

**Table 3:** Main postoperative outcomes of patients who underwent ALPPS.

Variable of Interest	Postoperative Major Complications		*p* Value
	No	Yes	
R0 resection margin *n* (%)	4 (13.3%)	26 (86.7%)	-
PHLF *n* (%)	25 (83.3%)	5 (16.7%)	-
Major complication after stage 1 *n* (%)	28 (93.3%)	2 (6.7%)	-
Major complication after stage 2 *n* (%)	19 (63.3%)	11 (36.7%)	-
ALPPS risk score before stage 2, median (range)	4.6 (3.1–7.3)	6.2 (3.5–9.6)	0.031
90-day mortality *n* (%)	23 (76.7%)	7 (23.3%)	-
ALPPS risk score before stage 2, median (range)	4.6 (3.1–7.9)	6.6 (4.2–9.6)	0.025

ALPPS: associating liver partition and portal vein ligation for staged hepatectomy; PHLF: posthepatectomy liver failure; SD: standard deviation.

**Table 4:** Perioperative data of patients who underwent ALPPS before and after modifications were introduced in 2016.

	Variables	Before 2016	After 2016	*p* Value
		Total (*n* = 14)	Total (*n* = 16)	
**Preoperative data**	Age (range)	63.5 (45–78)	68 (39–81)	0.541
	Female/male	4/10	3/13	0.525
	BMI (kg/m^2^) (range)	25.6 (19.0–29.0)	24.6 (19.7–29.7)	0.951
	Preoperative biliary stenting *n* (%)	1 (7.1%)	6 (37.5%)	0.049
	Tumor type *n* (%)			0.151
	PHCC *n* (%)	12 (85.7%)	10 (62.5%)	
	IHCC *n* (%)	2 (14.3%)	6 (37.5%)	
	ALPPS risk score before stage 1 (range)	2 (2–5)	2 (2–5)	0.552
**Stage 1**	Hepaticojejunostomy *n* (%)	8 (57.1%)	0 (0.0%)	-
	Operation time (minutes) (range)	337 (180–570)	240 (127–431)	0.013
**Interstage**	RLV after stage 1 (range)	20.5 (15–30)	23 (15.44–32)	0.712
	Major complication after stage 1 *n* (%)	1 (7.1%)	1 (6.3%)	0.956
	Median serum total bilirubin before stage 2 (mg/dL) (range)	1.4 (0.3–11)	1.0 (0.2–8)	0.294
	Median serum creatinine before stage 2 (g/L) (range)	0.7 (0.5–1.3)	0.7 (0.4–2.1)	0.667
	ALPPS risk score before stage 2 (range)	6.4 (3.5–9.8)	4.4 (3.1–6.7)	0.010
**Stage 2**	Operation time (minutes) (range)	97 (60–229)	207 (89–339)	0.002
	Hepaticojejunostomy *n* (%)	6 (42.9%)	16 (100%)	0.001
**Postoperative**	RLV after stage 2 (range)	47 (31–58)	47 (29.1–52)	0.754
	R0 resection margin *n* (%)	13 (92.9%)	14 (87.5%)	0.552
	PHBL *n* (%)	4 (28.6%)	1 (6.3%)	0.126
	Major complication after stage 2 *n* (%)	6 (42.9%)	5 (31.3%)	0.390
	PHLF *n* (%)	2 (14.3%)	3 (18.8%)	0.567
	30-day mortality *n* (%)	2 (14.3%)	0 (0%)	0.209
	90-day mortality *n* (%)	5 (35.7%)	2 (12.5%)	0.197

ALPPS: associating liver partition and portal vein ligation for staged hepatectomy; IHCC: intrahepatic cholangiocarcinoma; PHCC: perihilar cholangiocarcinoma; PHBL: posthepatectomy biliary leakage; PHLF: posthepatectomy liver failure; RLV: remnant liver volume.

## Data Availability

The data of the present study can be made available upon a logical request to the corresponding author and after evaluation of the request by the ethics committee of the university.

## Supplementary Materials

The following supporting information can be downloaded at: https://www.mdpi.com/article/10.3390/cancers15235613/s1, Figure S1. Three-year recurrence-free survival of patients who underwent ALPPS for PHCC. Figure S2. Three-year overall survival of patients who underwent ALPPS for PHCC.
