# Supplementary material for: Promising Outcomes of Modified ALPPS for Staged Hepatectomy in Cholangiocarcinoma
Source: Cancers (Basel). 2023 Nov 28;15(23):5613. doi: 10.3390/cancers15235613 (PMC10705795; doi:10.3390/cancers15235613)

Promising outcomes of modified associating liver partition and portal vein ligation for staged hepatectomy in cholangiocarcinoma

(Supplementary materials)

Figure S1. Three-year recurrence-free survival of patients who underwent ALPPS for PHCC.

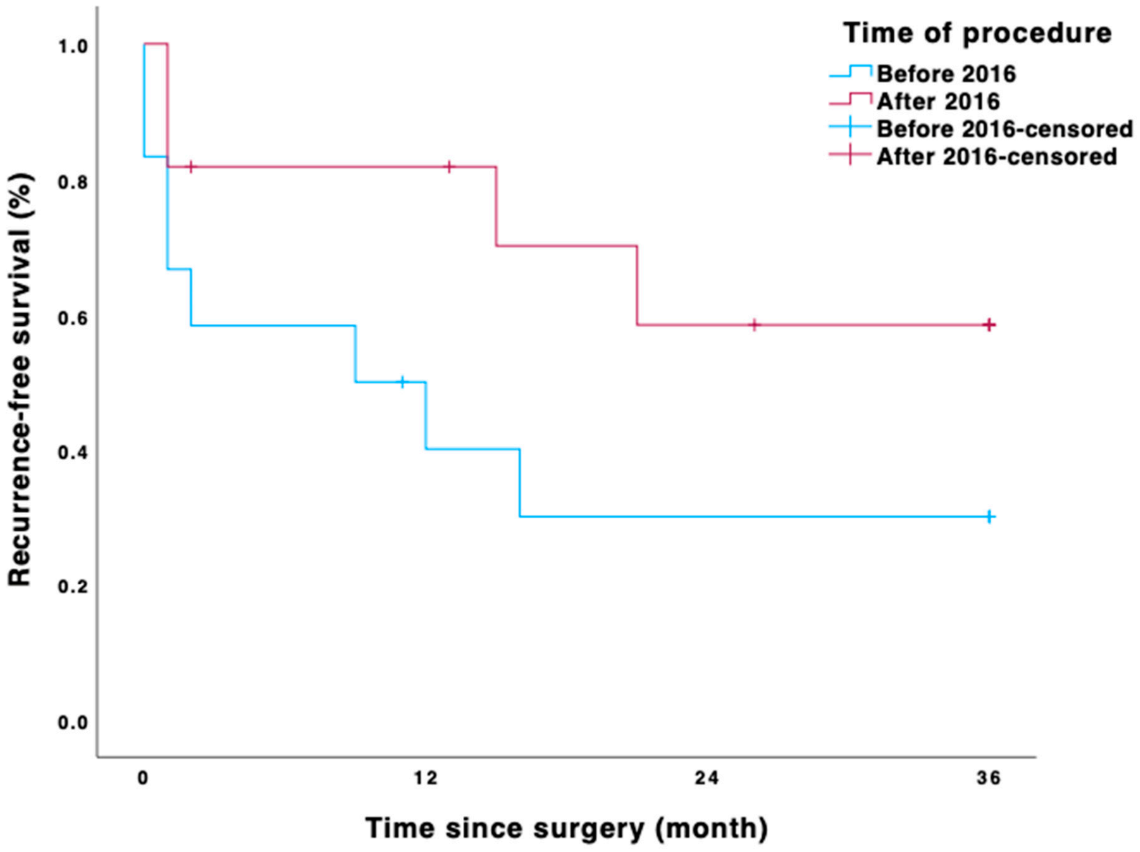

Figure S2. Three-year overall survival of patients who underwent ALPPS for PHCC.

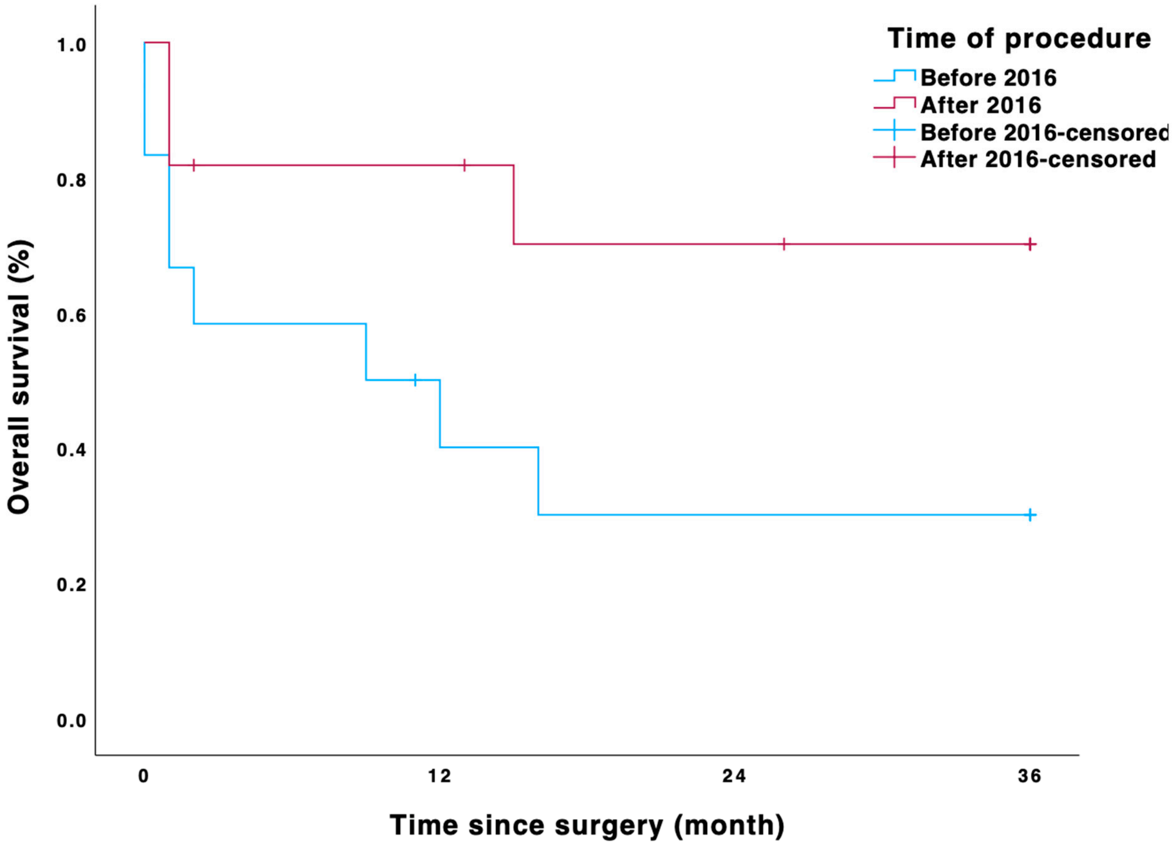

Supplement: Supplementary file 1 [file cancers-15-05613-s001.zip › cancers-2714050-supplementary.pdf]
